# Selective Blocking of TNF Receptor 1 Attenuates Peritoneal Dialysis Fluid Induced Inflammation of the Peritoneum in Mice

**DOI:** 10.1371/journal.pone.0163314

**Published:** 2016-10-18

**Authors:** Florian Kälble, Janine Damaske, Danijela Heide, Iris Arnold, Fabian Richter, Olaf Maier, Ulrich Eisel, Peter Scheurich, Klaus Pfizenmaier, Martin Zeier, Vedat Schwenger, Julia Ranzinger

**Affiliations:** 1 Department of Nephrology, University of Heidelberg, Heidelberg, Germany; 2 Institute of Cell Biology and Immunology, University of Stuttgart, Stuttgart, Germany; 3 Department of Molecular Neurobiology, Groningen Institute of Evolutionary Life Sciences, University of Groningen, Groningen, Netherlands; 4 Department of Nephrology, Klinikum Stuttgart, Stuttgart, Germany; National University Singapore Yong Loo Lin School of Medicine, SINGAPORE

## Abstract

Chronic inflammatory conditions during peritoneal dialysis (PD)-treatment lead to the impairment of peritoneal tissue integrity. The resulting structural and functional reorganization of the peritoneal membrane diminishes ultrafiltration rate and thereby enhances mortality by limiting dialysis effectiveness over time. Tumour necrosis factor (TNF) and its receptors TNFR1 and TNFR2 are key players during inflammatory processes. To date, the role of TNFR1 in peritoneal tissue damage during PD-treatment is completely undefined. In this study, we used an acute PD-mouse model to investigate the role of TNFR1 on structural and morphological changes of the peritoneal membrane. TNFR1-mediated TNF signalling in transgenic mice expressing human TNFR1 was specifically blocked by applying a monoclonal antibody (H398) highly selective for human TNFR1 prior to PD-treatment. Cancer antigen-125 (CA125) plasma concentrations were measured by enzyme-linked immunosorbent assay (ELISA). Western blot analyses were applied to determine TNFR2 protein concentrations. Histological staining of peritoneal tissue sections was performed to assess granulocytes within the peritoneal membrane as well as the content of hyaluronic acid and collagen. We show for the first time that the number of granulocytes within the peritoneal membrane is significantly reduced in mice pre-treated with H398. Moreover, we demonstrate that blocking of TNFR1 not only influences CA125 values but also hyaluronic acid and collagen contents of the peritoneal tissue in these mice. These results strongly suggest that TNFR1 inhibition attenuates peritoneal damage caused by peritoneal dialysis fluid (PDF) and therefore may represent a new therapeutic approach in the treatment of PD-related side effects.

## Introduction

PD is an effective renal replacement therapy and a well-established alternative to haemodialysis. Success as well as efficacy of this treatment is dependent on the integrity of the peritoneal membrane. Acute and chronic inflammatory conditions such as recurring peritonitis are causal for peritoneal damage [1]. Epithelial-mesenchymal transition (EMT) of human peritoneal mesothelial cells (HPMCs), specialized epithelial cells lining the peritoneal cavity, plays a central role in the onset and progression of peritoneal fibrosis during PD-treatment [2]. This process is causal for the failure of the peritoneal membrane function and the subsequent loss of ultrafiltration which accounts for the time restriction of PD-treatment.

During the last years, comprehensive knowledge concerning the functional role of TNF in health and disease has been gained. TNF has been identified as a central pathological mediator for a multitude of diseases such as tissue necrosis, fibrosis and EMT [3, 4]. To date, little is known about the role of TNF and its receptors, TNFR1 and TNFR2, in the pathology of peritoneal damage.

TNF-antagonists have been used with a remarkable clinical success in the treatment of autoimmune diseases. However, these drugs bind both soluble and membrane TNF not taking into account that TNF is of great importance in health and disease and that global inhibition of TNF coincides with several limitations such as the risk for severe infections. This awareness led to the development of TNF-receptor specific antagonistic antibodies such as H398 to selectively inhibit receptor-mediated TNF signalling [5–8]. In the present study, we analysed the effect of specifically blocking TNFR1 using H398 on peritoneal damage during PD-treatment. Due to the high selectivity of H398 for human TNFR1, we used transgenic mice expressing a chimeric hu/mTNFR1 [9].

## Materials and Methods

### Mice and Experimental Setup

Male huTNFR1 k/i mice of 10 to 13 weeks of age were used in all experiments. In these mice, mTNFR1 has been exchanged for a chimeric TNFR1 consisting of the extracellular domain of huTNFR1 and the transmembrane and intracellular domain of mTNFR1 by homologous recombination [9]. All strains were backcrossed to a C57BL/6 background a minimum of 14 generations. Mice were housed individually with a 12h/12h light/dark cycle and free access to food and water. All procedures in this study were approved by the Animal Care and Use Committees at the *Regierungspräsidium* Tübingen and Karlsruhe, Germany. For the experiments, mice were randomly allocated to 4 groups: i) untreated (n = 3); ii) Instillation of Dianeal 1,36% glucose (Baxter, Deerfield, USA) as PDF twice within 24h (n = 10); iii) H398 two hours prior to instillation of Dianeal 1,36% glucose twice within 24h (n = 8); iiii) H398 two hours prior to instillation of PBS twice within 24h (n = 7).

For the PD-experiment, mice received an intraperitoneal injection of H398 antibody (mouse monoclonal IgG2a [20mg/kg]), 1ml PDF Dianeal 1,36% glucose or PBS at 37°C under sterile conditions. After 24h of treatment, the experiment was terminated.

### Collection of Blood and Preparation of Peritoneal Tissue

Mice were anaesthetized by i.p. injection of 50 μl ketamine/xylariem (ketamine:xylariem 1:3). After blood collection via the abdominal aorta, perfusion was performed via the left ventricle with 30 ml sodium chloride solution and parietal peritoneal tissue was resected. For morphological analysis, tissue samples were fixed in zinc solution, embedded in paraffin and cut into 1μm thick tissue sections. For the preparation of protein lysates, tissue was immediately frozen in Tissue-Tek® O.C.T. at -80°C.

### Histological Staining

Tissue sections were deparaffinised by xylol and rehydrated. For histological staining, sections were stained with haematoxylin (according to Mayer T865) & eosin (X883, Carl Roth, Karlsruhe, Germany) using a standard protocol. For staining granulocytes, a Naphthol AS-D Chloroacetate Esterase kit (91C, Sigma-Aldrich, Taufkirchen, Germany) was used according to the manufacturer´s protocol. Hyaluronic acid (HA) was stained using a primary sheep polyclonal IgG antibody to HA (ab53842, 1:300, Abcam, Cambridge, UK). As secondary antibody, a rabbit anti-sheep IgG H&L (HRP) antibody (ab6747, 1:100, Abcam) was used. For visualization, a Peroxidase/DAB EnVision Detection kit (K4065, Dako Hamburg, Germany) was used according to the manufacturer´s protocol.

Images were taken using a Nikon Eclipse 80i microscope equipped with a Nikon Plan Fluor x20 NA 0.50 objective (Nikon, Düsseldorf, Germany). Image acquisition and processing were controlled by NIS Elements BR 3.0 software. Images were visualized and processes with Image J software (v.1.44o) (European Molecular Biology Laboratory).

### H398 Plasma Measurement

To determine H398 plasma concentrations, ELISA plates (Greiner, Frickenhausen, Germany) were coated with huTNFR1-Fc fusion protein at 1μg/ml in PBS and incubated at 4°C overnight. Residual binding sites were blocked with 2% skim milk powder in PBS for 2 hours. Then, plasma was added for 2 hours and bound proteins were detected by incubation with HRP-conjugated anti-mouse IgG for 1 hour followed by incubation with TMB substrate solution. Reaction was stopped by addition of 1M H_2_SO_4_ and the absorbance at 450nm was determined with an absorbance reader (Tecan, Männedorf, Switzerland) and data were analysed using the software Microsoft Excel and GraphPad Prism 4 (GraphPad, La Jolla, CA).

### CA125 Measurement

CA125 plasma concentration was measured using an ELISA kit for murine CA125 purchased from Cloud-Clone Corp. (Houston, USA) according to the manufacturer´s protocol.

### Western Blot Analysis

For Western Blot analyses, tissues (10mg each) were homogenized in RIPA-buffer (Invitrogen, Life Technologies, Darmstadt, Germany) containing protease inhibitors (Complete mini EDTA-free, Roche, Mannheim, Germany) using Tissuelyser LT (Qiagen, Hilden, Germany) following the manufacturer´s instructions at 50 Hz for 7 min. The total protein concentrations were determined using a BCA protein assay kit (Thermo Scientific, Langenselbold, Germany). Proteins were then resolved by SDS-PAGE, transferred to nitrocellulose membranes (Bio-Rad, München, Germany) and probed with the following primary antibodies 1 hour at room temperature: anti-TNFR2 (EPR1653, rabbit monoclonal IgG, 1:500, Abcam) and anti-vinculin (EPR8185, rabbit monoclonal IgG, 1:500, Abcam).

Detection was performed by using Western Breeze Chromogenic Immunodetection kits (Invitrogen, Life Technologies) according to the manufacturer´s instructions. Band density was analyzed by using Image J software (v.1.44o) and results were normalized to vinculin.

### Statistical Analyses

For quantification of granulocyte-numbers, three randomly chosen images for each animal were selected and the total number of granulocytes was counted using the particle analyzer plugin of Image J software (version 1.44o). The statistical data represent the mean value for each group. Graphs were generated with GraphPad Prism 5 software (Statcon, Witzenhausen, Germany). Error bars represent the standard error of the mean. The calculation of statistical significance was performed by the use of Student´s *t* test (Origin 6.1 software, OriginLab, Friedrichsdorf, Germany) in which the level of significance was set to *p<*0.05 and *p<*0.01.

## Results

**Fig 1:**
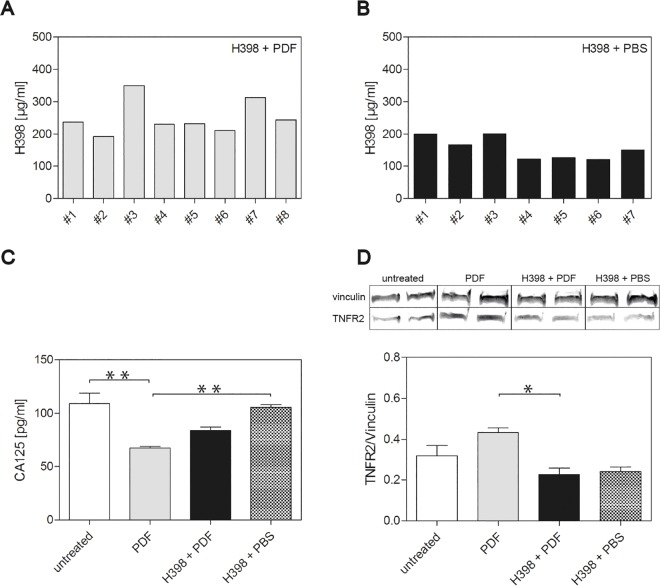
Impact of selective blockage of TNFR1 on CA125 plasma levels and TNFR2 protein amounts in the peritoneum. (A) and (B) H398 concentrations were measured by ELISA in plasma samples from mice pretreated with H398 2 hours before application of PDF (n = 8) (A) or PBS (n = 7) (B). (C) CA125 concentrations were measured by ELISA in plasma samples from untreated mice (white bar) (n = 3), mice treated with PDF (gray bar) (n = 10) and mice pre-treated with H398 2 hours before application of PDF (black bar) (n = 8) or PBS (shaded bar) (n = 7). (D) Western blot analyses of TNFR2 protein amounts in parietal peritoneum from untreated mice (white bar) (n = 3), mice treated with PDF (gray bar) (n = 10) and mice pretreated with H398 2 hours before application of PDF (black bar) (n = 8) or PBS (shaded bar) (n = 7). Protein levels were normalized to the corresponding vinculin loading control. For each experimental setup, two representative protein bands are depicted showing the vinculin band at 130 kDa and the TNFR2 band at 75 kDa corresponding to the intact receptor. Data are shown as means ± SEM, *p<0.05, **p<0.01 (t test in b and c).

It is well known that drugs are absorbed through diffusion into blood vessels upon intraperitoneal (IP) injection. To proof efficient IP injection of H398, we first investigated the plasma concentrations of H398 26 hours after the application. Fig 1 shows the concentrations of the antibody measured by ELISA in mice pre-treated with the antibody prior to injection of PDF (A) or PBS (B).

During PD-treatment, secreted or shed substances can be measured in the peritoneal effluent. In our study, we measured CA125 plasma concentrations by ELISA and showed a significant lower CA125 concentration in mice after application of PDF compared to untreated mice (Fig 1C, white and gray bar) and mice pre-treated with H398 and subsequent application of PBS (Fig 1C, gray and shaded bar). Remarkably, CA125 concentrations were less reduced in mice treated with H398 prior to the application of PDF compared to mice treated with PDF solely (Fig 1C, gray and black bar).

**Fig 2:**
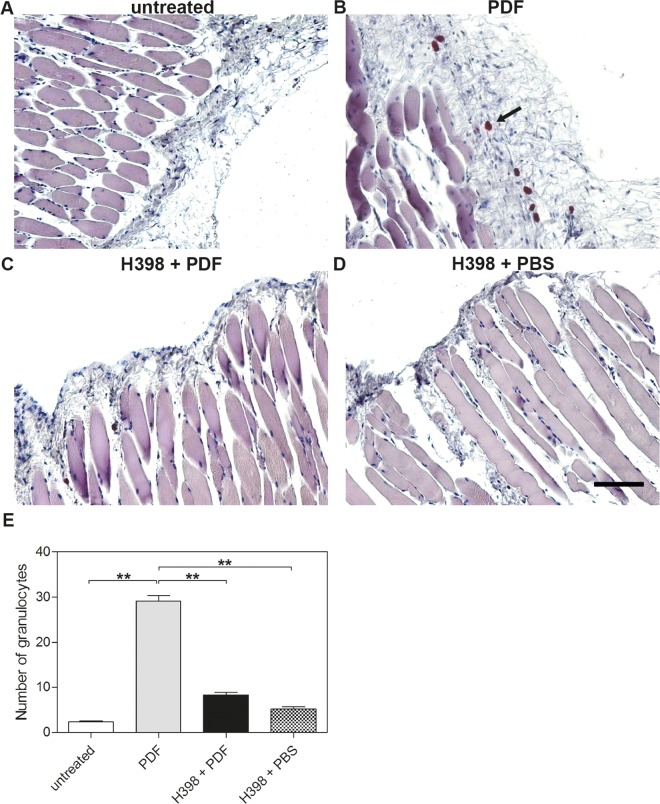
Selective inhibition of TNFR1 reduces the number of granulocytes within the peritoneum during PD-treatment in mice. Representative histological staining of parietal peritoneum showing granulocytes in large numbers in tissue from mice treated with PDF (B, arrow) compared to tissue from untreated mice (A) or mice pretreated with H398 2 hours before application of PDF (C) or PBS (D) (Scale bar: 100 μm). (E) Quantitative analyses of the number of granulocytes in the parietal peritoneum. Data are shown as means ± SEM, *p<0.05, **p<0.01.

In the context of TNF-initiated signalling it is known that TNFR1 and TNFR2 not only act independently but rather influence each other via crosstalk between the different signalling pathways [10]. In our study, Western blot analyses showed an increase in TNFR2 protein in the peritoneum of mice after application of PDF compared to untreated mice (Fig 1D, white and gray bar). However, treatment with H398 prior to the application of PDF significantly decreased TNFR2 protein amounts (Fig 1D, gray and black bar). Treatment with H398 prior to application of PBS resulted in comparatively low TNFR2 protein amounts (Fig 1D, black and shaded bar). To elucidate whether selective inhibition of TNFR1 with H398 affects inflammatory reactions of the peritoneum during PD-treatment, we performed a Leder stain on tissue sections of parietal peritoneum from untreated mice (Fig 2A), after application of PDF (Fig 2B), pre-treated with H398 (Fig 2C) and pre-treatment with H398 prior to application of PBS (Fig 2D) and assessed Naphthol AS D chloroacetate esterase-positive cells of granulocytic lineage within the tissue.

We found infiltration of these cells in the peritoneal membrane of mice treated with PDF in large numbers (Fig 2B, arrow and Fig 2E). The number of these cells was significantly reduced when mice were pre-treated with H398 (Fig 2C and 2E). The injection of PBS did not lead to the infiltration of granulocytes in the peritoneal membrane (Fig 2D and 2E).

**Fig 3:**
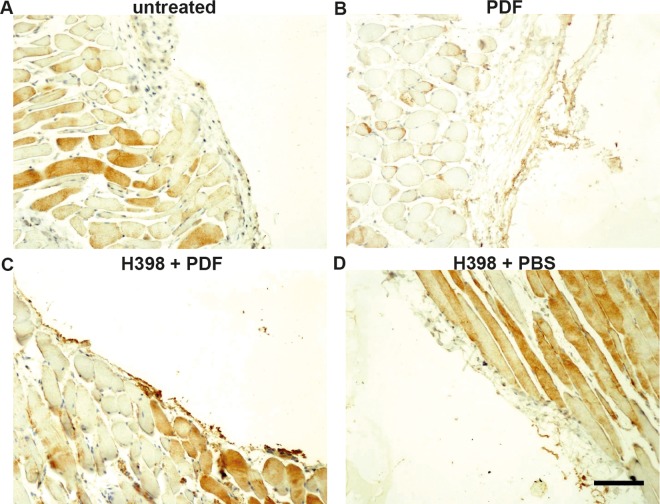
Impact of TNFR1 blockage on hyaluronic acid (HA) content in the peritoneum. Representative immunohistochemical staining of parietal peritoneum tissue sections displaying HA in brown color (A-D). HA is dramatically reduced in the peritoneum from mice treated with PDF (B) compared to tissue from untreated mice (A) and mice pretreated with H398 2 hours before application of PDF (C) or PBS (D) (Scale bar: 100μm).

Hyaluronic acid (HA) is a glycosaminoglycan present in the interstitium of the peritoneal membrane and is a major component of the glycocalyx forming a protective barrier around mesothelial cells [11]. The glycocalyx has recently been associated with the recruitment of leukocytes to sites of inflammation as well as reparative processes upon tissue injury [12, 13]. Moreover, dialysis patients with high levels of inflammation have a more damaged glycocalyx barrier [14]. In light of these findings, we performed a HA staining on parietal peritoneum tissue sections displaying HA in brown colour (Fig 3). We found striking differences in the HA content of the respective peritoneal tissue displayed by the varying colour intensities (Fig 3A–3D). Peritoneal tissue obtained from mice after PDF-application showed a denotative reduction of HA compared to tissue from untreated mice (Fig 3A and 3B). By contrast, tissue from mice pre-treated with H398 showed an equivalent colour intensity for HA compared to tissue from untreated mice (Fig 3A and 3C). The same applies to pre-treatment with H398 prior to application of PBS (Fig 3D).

**Fig 4:**
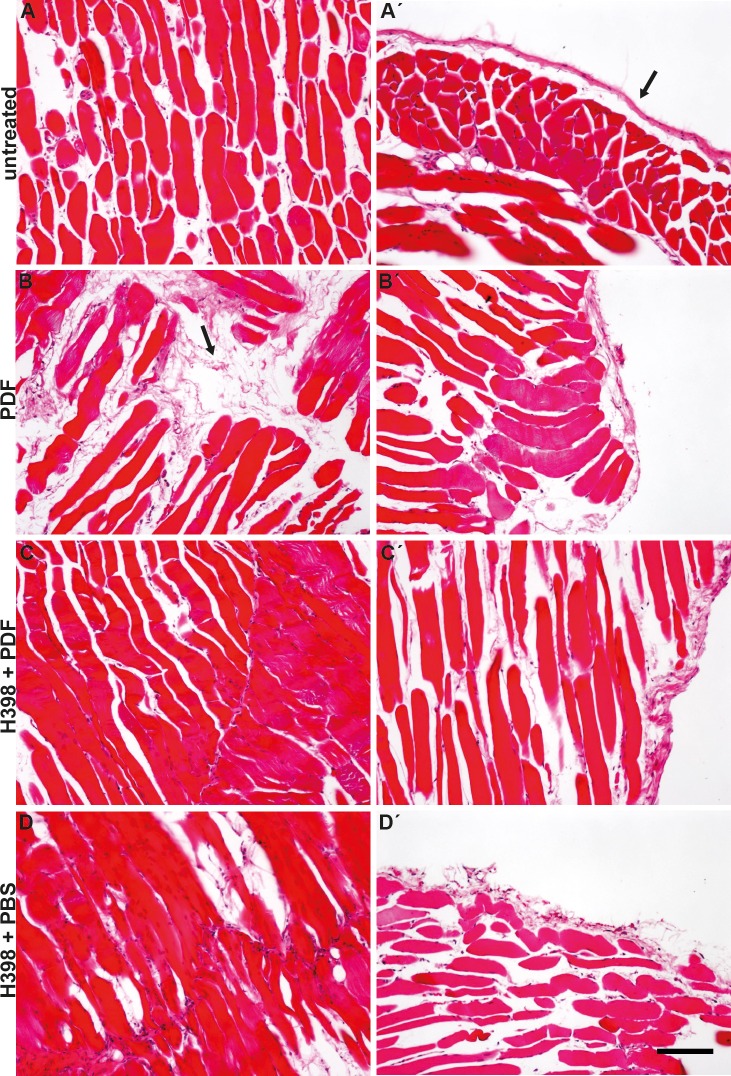
Inhibition of TNFR1 signalling by H398 reduces PDF caused gain of collagen in the peritoneum. Representative histological staining of collagen in parietal peritoneum. Compared to tissue from untreated mice (A), collagen is highly expressed in the submesothelial layer of mice treated with PDF (B, arrow). An increased accumulation of collagen is also displayed along the mesothelial cell layer (B´, arrow) in comparison to the control (A´, arrow). In mice pre-treated with H398 2 hours before application of PDF or PBS, collagen is less expressed in both the submesothelial layer (C and D) and along the mesothelial cell layer (C´ and D´) (Scale bar: 100μm).

Long term PD-treatment leads to structural changes in all parts of the peritoneal membrane, including an increase in fibrotic tissue, in fact both subepithelial and interstitial [15]. The role of transforming growth factor beta 1 (TGF-β1) in the development of peritoneal fibrosis in PD-patients is well established [16]. However, the role of inflammatory cytokines in the induction of fibrosis is not understood. To elucidate a possible role of TNFR1 in the onset of peritoneal fibrosis, we displayed collagen in parietal peritoneum tissue sections with and without selective inhibition of TNFR1 via haematoxylin & eosin staining (Fig 4). Tissue sections from mice treated with PDF show a disturbed cell layer lined with an accumulation of collagen as well as a massive increase of collagen in the interstitium (Fig 4B and 4B´ arrow) compared to tissue sections from untreated mice or mice treated with H398 followed by application of PBS (Fig 4A, 4A´ and 4D and 4D´). Interestingly, this gain in collagen was substantially attenuated when TNFR1 had been blocked with H398 prior to application of PDF (Fig 4C and 4C´).

## Discussion

In the present study, we specifically blocked TNFR1-mediated TNF responses using the monoclonal antibody H398 in an acute PD-mouse model to investigate a pathogenic role of TNFR1 during PD-induced inflammation and potential therapeutic activity of a TNFR1 antagonistic antibody. To better refer to the clinical situation and taking advantage of the selectivity of H398 for human TNFR1, we used huTNFR1 k/i transgenic mice for these investigations [9]. We show that blocking of TNFR1 in these mice not only has a strong impact on PD-related inflammatory reactions of the peritoneal membrane, but also on plasma concentrations of CA125, as well as the content of HA and collagen within the peritoneal tissue. For many years, CA125 has been used as a biomarker for mesothelial cell mass in patients undergoing peritoneal dialysis treatment. However, over time, the interpretation of CA125 levels has become a subject of considerable discussion [17]. In this context, recent research showed that a decline in dialysate CA125 levels may indicate mesothelial cell damage and/or a risk to develop encapsulating peritoneal sclerosis (EPS) [18]. Furthermore, the question has been raised, whether this molecule could represent a surrogate marker of inflammation in peritoneal dialysis effluent. The results presented in our manuscript strongly support the hypothesis that CA125 concentrations correlate with inflammatory conditions during peritoneal dialysis.

Moreover, we showed that intraperitoneal injection of H398 two hours prior to the application of PDF significantly reduces the presence of cells of granulocytic lineage within the peritoneal tissue pointing to an attenuation of inflammatory reactions. Given this finding, the question arises, whether TNFR1 guides cells of the immune system to sites of inflammation within the peritoneum. Furthermore, the finding that the amount of collagen in the interstitium and along the peritoneal membrane is strongly reduced upon blocking of TNFR1 strongly points to an involvement of this receptor in the development of structural and morphological changes of the peritoneal membrane which may further lead to the onset of EMT. This hypothesis is strongly supported by a previous study showing that compared to wild-type mice, collagen deposition is greatly attenuated and markedly fewer CD34^+^CD45^+^ cells are present in the heart of TNFR1-KO mice upon Angiotensin-II infusion [19].

In the last years evidence accumulated that TNFR2-mediated signalling provides protection in several degenerative disorders [20]. In our study, we show that TNFR2 protein levels are strongly increased in peritoneal tissue from mice treated with dialysis solution compared to tissue from untreated mice as well as mice pre-treated with H398. These significant differences in TNFR2 protein amounts depending on the ability of TNFR1 to signal under conditions when cells are exposed to non-physiological strains like dialysis solutions argues for a so far unknown function of TNFR2 in the peritoneum. Whether these increased TNFR2 protein levels can be attributed to the infiltrated immune cells expressing TNFR2 in high numbers or the upregulation of TNFR2 in mesothelial cells due to PDF causing an inflammatory environment is completely unknown at the moment.

Over the years, it has become obvious that selective inhibition of TNFR1 can be superior to global blockage of TNF in several models of degenerative diseases [21, 22]. In the present study, we preclinically investigated a potential therapeutic effect of H398 on inflammatory reactions and structural changes of the peritoneum in a transgenic mouse model during PD-treatment and provide evidence that H398 attenuates inflammation and structural damage of the peritoneum in these mice. Considering these findings, the application of TNFR1 antagonists in PD-treatment could help attenuating peritoneal membrane damage, and therefore open new therapeutic options to preserve long term peritoneal membrane function.
